# Supplementary material for: Menstrual cycle effects on cognitive performance: A meta-analysis
Source: PLoS One. 2025 Mar 17;20(3):e0318576. doi: 10.1371/journal.pone.0318576 (PMC11913300; doi:10.1371/journal.pone.0318576)
Supplement: Attachment — Submitted filename: Response to Reviewers.docx [file pone.0318576.s012.docx]

Dear Dr. Swelum,

We thank you for the opportunity to revise our manuscript for further consideration at PLOS ONE.

Below, we address each of the issues pointed out by the reviewers as well as required by the journal.

Yours,

Daisung Jang

Melbourne Business School

Journal Requirements:

When submitting your revision, we need you to address these additional requirements.

1. Please ensure that your manuscript meets PLOS ONE's style requirements, including those for file naming. The PLOS ONE style templates can be found at

<https://journals.plos.org/plosone/s/file?id=wjVg/PLOSOne_formatting_sample_main_body.pdf> and

[https://journals.plos.org/plosone/s/file?id=ba62/PLOSOne_formatting_sample_title_authors_affiliations.pdf](https://c05y1x9s.r.us-east-2.awstrack.me/L0/https:%2F%2Fjournals.plos.org%2Fplosone%2Fs%2Ffile%3Fid=ba62%2FPLOSOne_formatting_sample_title_authors_affiliations.pdf/1/010f0192650751ca-58a40c1d-b49f-43ed-80ae-f0cf33d0f52e-000000/JeQJfEm25wb50Zy8V6orQn5WeKw=179)

**Response:** We have followed PLOS ONE’s style requirements in the revision.

2. We note that you have referenced (unpublished) on page 10, which has currently not yet been accepted for publication. Please remove this from your References and amend this to state in the body of your manuscript: (ie “Bewick et al. [Unpublished]”) as detailed online in our guide for authors

[http://journals.plos.org/plosone/s/submission-guidelines#loc-reference-style](http://c05y1x9s.r.us-east-2.awstrack.me/L0/http:%2F%2Fjournals.plos.org%2Fplosone%2Fs%2Fsubmission-guidelines%23loc-reference-style/1/010f0192650751ca-58a40c1d-b49f-43ed-80ae-f0cf33d0f52e-000000/oLzGnsp0-rq9e52dr09PpYWtWgA=179)

**Response:** On page 10, we discuss our methods for the meta-analysis, which involves potentially including results from unpublished papers. We do not cite an unpublished paper in that section or elsewhere in the manuscript.

3. Please respond by return e-mail with an updated version of your manuscript to amend either the abstract on the online submission form or the abstract in the manuscript so that they are identical. We can make any changes on your behalf.

**Response:** Below is the version of the abstract for both the online submission form and the manuscript:

Does a woman’s cognitive performance change throughout her menstrual cycle? Menstruation continues to be a taboo topic, subject to myths about how it affects women. Despite the considerable number of empirical studies, there have been few quantitative summaries of what is known. To address this gap, we conducted a meta-analysis of cognitive performance across the menstrual cycle, including the domains of attention, creativity, executive functioning, intelligence, motor function, spatial ability, and verbal ability. We included studies that measured women’s performance at specific points in the cycle for tasks that have objectively correct responses. Our analysis examined performance differences across phases using Hedges’ g as the effect size metric. Across 102 articles, N = 3,943 participants, and 730 comparisons, we observe no systematic robust evidence for significant cycle shifts in performance across cognitive performance. Although two results appeared significant with respect to differences in spatial ability, they arise from a large number of statistical tests and are not supported in studies that use robust methods to determine cycle phase. Through the use of Egger’s test, and examination of funnel plots, we did not observe evidence of publication bias or small-study effects. We examined speed and accuracy measures separately within each domain, and no robust differences across phases appeared for either speed or accuracy. We conclude that the body of research in this meta-analysis does not support myths that women’s cognitive abilities change across the menstrual cycle. Future research should use larger sample sizes and consistent definitions of the menstrual cycle, using hormonal indicators to confirm cycle phase.

4. As required by our policy on Data Availability, please ensure your manuscript or supplementary information includes the following:

A numbered table of all studies identified in the literature search, including those that were excluded from the analyses.

**Response:** S10 Table contains a list of every paper included in the analyses. A list of every paper that was initially considered was not possible because we only retained bibliographic records of papers that passed a first relevance check for study domain. S11 Table also contains a list of papers that were excluded from the analyses that passed the relevance test but upon closer inspection did not contain sufficient data.

For every excluded study, the table should list the reason(s) for exclusion.

**Response:** We now better document the process by which papers were selected and updated Figure 1 to reflect this. We did not previously report the screening step prior to final analyses and do so in the revised version. S11 Table contains a list of papers that were excluded at the third selection because on closer inspection, they did not contain sufficient data, the measure of cognition was not classifiable, or the study did not operationalize the menstrual cycle in a way that could be used in the meta-analysis.

If any of the included studies are unpublished, include a link (URL) to the primary source or detailed information about how the content can be accessed.

**Response:** All manuscripts were either published or available on a dissertation database. We note this on page 11.

A table of all data extracted from the primary research sources for the systematic review and/or meta-analysis. The table must include the following information for each study:

Name of data extractors and date of data extraction

**Response:** The first author extracted all of the data. However, because specialized software was not used to conduct meta-analyses, date of data extraction was not recorded. We now note this on page 13.

Confirmation that the study was eligible to be included in the review.

**Response:** We note on page 15 that all studies included in the data analyses were eligible to be included in the review.

All data extracted from each study for the reported systematic review and/or meta-analysis that would be needed to replicate your analyses.

**Response:** All data required to replicate the study are presented in S1 Table. We note this on page 15.

If data or supporting information were obtained from another source (e.g. correspondence with the author of the original research article), please provide the source of data and dates on which the data/information were obtained by your research group.

**Response:** Data obtained from correspondence with an author of the original research article are recorded and dates when data were obtained are recorded in the notes of S1 Table.

If applicable for your analysis, a table showing the completed risk of bias and quality/certainty assessments for each study or outcome. Please ensure this is provided for each domain or parameter assessed. For example, if you used the Cochrane risk-of-bias tool for randomized trials, provide answers to each of the signaling questions for each study. If you used GRADE to assess certainty of evidence, provide judgements about each of the quality of evidence factor. This should be provided for each outcome.

**Response:** On page 30, we report publication bias through the examination of a funnel plot. We also conducted a formal test of publication bias, namely Egger’s test. It showed that funnel plot asymmetry was not significant (b = 0.025, t = -1.250, df = 1163, p = 0.212), indicating there was no evidence for small-study or publication bias.

An explanation of how missing data were handled.

**Response:** We did not attempt to impute any missing data. We note this on p. 15.

This information can be included in the main text, supplementary information, or relevant data repository. Please note that providing these underlying data is a requirement for publication in this journal, and if these data are not provided your manuscript might be rejected.

Reviewers' comments:
Reviewer's Responses to Questions

5. Review Comments to the Author

Please use the space provided to explain your answers to the questions above. You may also include additional comments for the author, including concerns about dual publication, research ethics, or publication ethics. (Please upload your review as an attachment if it exceeds 20,000 characters)

Reviewer #1: The meta-analysis presented by the authors demonstrates a clear and well-structured analysis of the available literature. It was written clearly, with standard English and a neutral tone.

The author did not mention about the type of oral contraceptive pills. Combined oral contraceptive pills mimic the natural cycle and progesterone-only pills include progesterone and suppress oestrogen levels. Without specifying which type of oral contraceptives were used in the included studies, it becomes difficult to interpret the findings in a meaningful way, especially when evaluating outcomes related to hormonal changes. I believe it would be better to clarify which type of oral contraceptive pills were used in the meta-analysis.

**Response:** Thank you for your thoughtful comments. Unfortunately, of the twelve studies that recruited women using oral contraceptives (OC), seven did not report the type of oral contraceptive used. Of the five that did report OC types used by women, four did not report results by OC type. Within these samples, authors reported different kinds of OC being used by the recruited women. Only one study reported results by OC type. This made it impossible to meta-analyze outcomes related to specific OC formulation. We recognize this as a limitation of the study on page 37.

Why the authors felt aggregating the follicular, luteal and premenstrual phases was required? Follicular and luteal stages are completely two different hormonal stages of the menstrual cycle. Five-phase analysis is meaningful and it correlates with the physiological changes and hormonal status of the body.

**Response:** We thank the reviewer for this point and they are correct in that the five phase definition of the cycle appropriately separates the phases of the cycle. Thus, we focus our primary attention on analyses based on the five phase definition. The three phase definition does indeed aggregate different hormonal stages and we recognize that in the earlier version we did not provide sufficient justification for the aggregation. We chose to isolate the menstrual phase because it is readily detectable, and thus most accurately identified. We also isolated the ovulation phase because it is critical to reproduction and thus has theoretical implications. It is also detectable through multiple physiological tests (LH surge, body temperature shift). We sought to contrast these phases to the remainder of phases that may be less detectable, especially with respect to self-report or particular hormone markers. We explain this logic on pages 17-18 and appreciated the opportunity to clarify further the rationale behind the aggregation.

The authors have clearly outlined the methodology, and their assessment of publication bias and limitations is comprehensive. The conclusion of the study would guide the policies and social justice for women.

Overall, this meta-analysis is well-executed, making it accessible and informative for readers.

**Response:** We thank you for your helpful comments and for the opportunity to further strengthen the manuscript. Further, we appreciate your point about the value of this research for social justice.

Reviewer #2: I believe that the authors of this Systematic Review and meta-Analysis put much efforts in data collection, articles selection based on the outline measures, in acompay with statistical analysis to support the manuscript concepts.

However, some sections (methods, discussion and limitations sections) in this manuscript require certified English review, and Proof-Reading tools use to check again at grammer level.

**Response:** We thank you for your constructive comments and we have edited the manuscript with the assistance of a native English speaker.

Regarding the "Literature search strategy" at Methods section, the authors must clarify:
- The time limit for the collected raw articles /researches (i.e., up to Month, year) at the conducted search, and emphasize if being applied differently in one or more databases.

**Response:** We apologize for the misunderstanding and all databases we examined were searched for records as recent as January 2024. We clarify this on p. 11.

- the authors stated that 102 articles - met criteria for inclusion - out of 11,759 potential papers being collected were selected WITHOUT specialized software to conduct the literature search, and this cannot be rational due to absolute risk of selection bias and higher rates of removing possibly included articles. Besides, a de-duplication of all articles must be applied before screening by two reviewers. Thus, there MUST be a use of a validated reference software to handle and facilitate previously essential steps for articles screening and inclusion.

**Response:** We clarify our methodology further on page 11. Although we did not use specialized software to conduct a systematic search, we used citation management software (Zotero) to keep track of articles, both in terms of the originating database and in terms of managing duplicate entries. We apologize for the misunderstanding based on how we described the procedure.

- Please note that "Not assessing individual studies for risk of bias, as it was not a part of the study design" is not accepted, because non-reporting biases lead to bias due to missing evidence in a systematic review. As well, Meta-analyses are at risk of bias due to missing evidence when results of some eligible studies are unavailable because of the P value, magnitude or direction of the results. Thus, the authors must provide a list of those 102 included studies, their design and corresponding Risk of bias assessment for each by referring the modified Cochrane Collaboration tools.

**Response:** All articles analyzed in the meta-analysis are listed in S10 Table in the supplementary materials, and this is now mentioned on p. 30. Regarding the design of individual studies (e.g., methods used to track cycle, cross-sectional / longitudinal design / study site), these are listed on a per-sample basis in S1 Table. We analyze formally the risk of missing evidence (i.e., file drawer problem) through the examination of a funnel plot and through the use of Egger’s test for plot asymmetry (page 30), which revealed no evidence of publication bias.

Reviewer #3: Dear Author,
Thank you very much for your contribution to science. The effort needed and methodology requires a very high training and time very valuable.
Going through the document, the style, redaction and contents are easy to read and understand. The explanation of limitations and methodology are complete and adapted to the type of study. It is well addressed that some steps of a systematic review and meta-analysis are not performed (e.g. study protocol, evaluation of the risk of bias in the studies included) and such there are some limitations pointed out in the study. I would like to address some points so it is considered to add some comments on this:

**Response:** We thank you for your kind words and constructive comments.

- More than 50% of studies included were published more than 20 years ago. Is it any specific support to consider they are comparable to more current situations? Social, cultural, environmental issues are very likely different, and so they can affect cognitive abilities as well.

**Response:** We appreciate this thoughtful suggestion and in response engaged in additional analyses that examined studies conducted 20 years ago vis-a-vis those published more recently. At the end of this letter we provide a summary table analogous to Table 2 presented in the manuscript.

For studies conducted between 2004 and 2024, there were six significant differences in the overall and hormone confirmation subsets. Of those differences only one was adequately powered (i.e., sample size N = 273 or greater; see our response to reviewer 4, point 1), and involved samples that used hormone confirmation tests. However, when corrected for multiple comparisons, the p-value was no longer significant for that comparison (Phase 1 vs. remainder of phases for spatial ability accuracy).

For studies conducted in 2003 and earlier, there were more significant differences. They were observed in the domains of attention, memory, spatial ability, and verbal ability. Differences among hormone confirmation studies were observed for memory, with lower performance in the menstrual vs. luteal phase, and lower performance in premenstrual vs. follicular phase. However, none of those differences were detected in samples that were adequately powered.

Given that a persistent issue present in the literature is the use of small sample size, we conclude that the literature has not accumulated enough data to meaningfully compare social, cultural, and environmental issues. We hope that noting this in the limitations (pages 35-36) will spur additional research to better understand these factors.

- The range of mean ages in the studies included is really wide. Considering menstrual cycle varies across ages and occurrence of pregnancies, proximity to menarche or to perimenopause, among other situations, this constitutes a limitation too.

**Response:** We agree that it is important to consider this factor when interpreting the results and make note of this as a limitation on page 37, discussing that future research should formally study the effect of age as well as proximity to pregnancy, menarche, and perimenopause when considering menstrual cycle effects.

As I mentioned before, I consider limitations are generally well addressed and the perspective of using this knowledge to build new and better scientific production is appropriately indicated.

**Response:** Thank you for your positive feedback and for recognizing the efforts we've made in addressing the limitations. We appreciate your acknowledgment of our work’s potential to contribute to future scientific advancements.

Reviewer #4: Congratulations to the authors for developing a manuscript that addresses a significant research gap in the field of women’s health. The study presents a clear and well-defined research objective and draws on a substantial volume of data, which holds great potential for generating meaningful conclusions. To further elevate the quality of the manuscript, I would like to offer the following suggestions for improvement.

**Response:** We appreciate these kind words about the paper.

1. The manuscript lacks consistency in how menstrual cycle phases are defined across different studies included in the meta-analysis. Relying on self-reports for phase identification can introduce bias, especially if hormonal confirmation is absent. It may be beneficial to provide a more consistent operational definition for menstrual cycle phases across studies. If hormonal confirmation is not available for all studies, I recommend including a section on how the variability in phase definition was handled and consider conducting sensitivity analyses to assess the impact of self-reported phase data. I believe this will strengthen the reliability of the results.

**Response:** Thank you for this point, which shows that our description of the analytic approach needs to be clearer. We agree that a consistent operational definition is necessary to compare across studies, and this is an ongoing concern in the literature [1]. Thus, we attempted to standardize cycles by using a previously established range of cycle dates as a benchmark [2]. This involved coding cycles not necessarily according to cycle phase definitions reported in the study under examination, but assigning them to those matching the established range. We also conducted separate analyses only for studies that use hormone confirmation methods, because they are the most accurate in determining cycle phase [3]. However, because cycle phase operationalization in terms of self-reported days was a considerable portion of the data, we conducted sensitivity tests by conducting analyses on studies that overlapped 80% with our cycle phase definitions (pages 17-18). These results are shown in S2-S9 Tables. In the revision, we have worked to highlight these features of the analytical approach, on pages 13-14.

2. There is no clear indication that a power calculation was performed to ensure that the meta-analysis could detect small or moderate effects across the cognitive domains examined. To increase the robustness of the findings, I suggest including a formal power analysis. This would help determine whether the study was adequately powered to detect small or moderate effects across all cognitive domains.

**Response:** The revised manuscript now includes a power analysis using the metapower package (version 0.2.2) with the R statistical software language [4]. The package was developed following the power analysis approach in meta-analyses suggested by past research [5,6]. We used an estimated small-to-medium effect size of the comparisons across phases of the menstrual cycle (Hedge’s g = .35) and the average participant sample size of the studies in our meta-analysis (N = 39) to determine the number of studies needed to detect the effects [7,8]. The analysis suggests that at least seven studies are required to achieve 80% power at an α of .05. The results caution in interpreting results for estimates involving fewer than 273 participants. We include this power analysis on page 31.

3. The discussion of non-significant results is somewhat lacking in depth. While null findings are reported, the manuscript could benefit from a more comprehensive discussion of the possible reasons behind these results. I recommend expanding the discussion around the non-significant findings, especially by considering methodological differences between studies, small sample sizes, or other factors that could explain the lack of significant results. Providing more context will give readers a better understanding of these findings and their implications.

**Response:** We appreciate this point and agree that strictly speaking it is not possible to prove a null or show definitive evidence of non-significance using frequentist statistical methods. We follow your suggestion to speculate about the lack of significant results on pages 33-34. Among other reasons, the null finding could be attributed to studies being conducted in developed countries with a high standard of living. This affords women with health education, access to hygiene products, and high levels of nutrition. We speculate that these social and environmental effects could moderate the effects of hormonal shifts.

4. The manuscript does not adequately address how confounding variables (such as age, education, and socioeconomic status) were controlled in the meta-analysis or the original studies. It would be meaningful to include a section discussing whether the included studies controlled for specific confounders. If this information was not available, I suggest addressing the potential impact of these factors in the limitations section to strengthen the transparency of the analysis.

**Response:** We thank you for this thoughtful comment. We now note that potential confounding variables were not controlled in the original studies. Often sample sizes are small (Mean N = 38.66). This would have meant that controlling for confounding variables would not have yielded useful conclusions due to a lack of statistical power—to detect a correlation of .30 at 80% power, a sample size of 84 is required in an individual study, far beyond the average sample size. The reviewer’s important point represents an opportunity for future research as we detail on page 36.

5. The two statistically significant results in spatial ability may be at risk of being over-interpreted, given the large number of comparisons conducted. Given the multiple comparisons made in this study, it would be prudent to apply more stringent correction methods (e.g., Bonferroni or FDR correction) to ensure the robustness of significant findings. Additionally, a more conservative interpretation of these results may help contextualize them more appropriately.

**Response:** We agree that the significant results may be overinterpreted given the large number of statistical tests we conduct and have applied Bonferroni corrections to the p-values of the significant results. Few of the differences were robust to corrections and none were robust to both Bonferroni corrections and use of more rigorous methodologies. We have updated the results and discussion sections to match this observation.

6. The results section could be clearer. While it’s written comprehensively, it may be overwhelming for readers, and the key findings may be buried in the details. I recommend summarizing the key results more clearly in the main text, possibly by creating a concise summary table or figure. Detailed data can be moved to supplementary materials, allowing readers to more easily follow the central findings.

**Response:** Thank you for the suggestion to provide a summary figure to accompany a lengthy results section. We have added a table (Table 2) to communicate results in a streamlined way.

7. The manuscript touches on the cultural and societal implications of menstrual cycle research but does not explore these in enough depth, particularly regarding the influence of external factors on women’s cognitive performance. I encourage expanding the discussion of how cultural and societal factors might influence women’s cognitive performance during their menstrual cycle. Exploring these broader implications would enrich the manuscript’s relevance and offer valuable context to the findings.

**Response:** As in our response to your third point, we now discuss the social and environmental factors to a greater degree on pages 35-36. Although we can speculate about these important factors, unfortunately, there is insufficient data to address this in the literature to date. We indeed recognize that external factors can influence psychological response and that future research will be needed to address this. We discuss this as a limitation on page 36.

**References**

1.	Jang D, Elfenbein HA. Menstrual cycle effects on mental health outcomes: A meta-analysis. Archives of Suicide Research. 2019; 312–332. doi:10.1080/13811118.2018.1430638

2.	Riley JL, Robinson ME, Wise EA, Price DD. A meta-analytic review of pain perception across the menstrual cycle. Pain. 1999;81: 225–235. doi:10.1016/S0304-3959(98)00258-9

3.	Wood W, Kressel L, Joshi PD, Louie B. Meta-Analysis of Menstrual Cycle Effects on Women’s Mate Preferences. Emotion Review. 2014;6: 229–249. doi:10.1177/1754073914523073

4.	Griffin JW. Calculating statistical power for meta-analysis using metapower. The Quantitative Methods for Psychology. 2021;17: 24–39. doi:10.20982/tqmp.17.1.p024

5.	Hedges LV, Pigott TD. The power of statistical tests in meta-analysis. Psychol Methods. 2001;6: 203–217.

6.	Hedges LV, Pigott TD. The power of statistical tests for moderators in meta-analysis. Psychol Methods. 2004;9: 426–445. doi:10.1037/1082-989X.9.4.426

7.	Cohen J. Statistical Power Analysis for the Behavioral Sciences. 2nd ed. New York: Routledge; 1988. doi:10.4324/9780203771587

8.	Cohen J. A power primer. Psychol Bull. 1992;112: 155–159. doi:10.1037//0033-2909.112.1.155

Analyses of studies published in 2003 and earlier

Analyses of studies published in 2004 and later
